# Integrated analysis of the transcriptome and metabolome reveals the molecular mechanism regulating cotton boll abscission under low light intensity

**DOI:** 10.1186/s12870-024-04862-7

**Published:** 2024-03-12

**Authors:** Ning Zhao, Zhao Geng, Guiyuan Zhao, Jianguang Liu, Zetong An, Hanshuang Zhang, Pengfei Ai, Yongqiang Wang

**Affiliations:** 1 Institute of Cotton, Hebei Academy of Agriculture and Forestry Sciences, Key Laboratory of Cotton Biology and Genetic Breeding in Huanghuaihai Semiarid Area, Ministry of Agriculture and Rural Affairs, Shijiazhuang, P.R. China; 2 College of Food Science and Biology, Hebei University of Science and Technology, Shijiazhuang, P.R. China

**Keywords:** Cotton, Abscission, Transcriptome, Metabolome, Molecular mechanisms

## Abstract

**Background:**

Cotton boll shedding is one of the main factors adversely affecting the cotton yield. During the cotton plant growth period, low light conditions can cause cotton bolls to fall off prematurely. In this study, we clarified the regulatory effects of low light intensity on cotton boll abscission by comprehensively analyzing the transcriptome and metabolome.

**Results:**

When the fruiting branch leaves were shaded after pollination, all of the cotton bolls fell off within 5 days. Additionally, H_2_O_2_ accumulated during the formation of the abscission zone. Moreover, 10,172 differentially expressed genes (DEGs) and 81 differentially accumulated metabolites (DAMs) were identified. A KEGG pathway enrichment analysis revealed that the identified DEGs and DAMs were associated with plant hormone signal transduction and flavonoid biosynthesis pathways. The results of the transcriptome analysis suggested that the expression of ethylene (ETH) and abscisic acid (ABA) signaling-related genes was induced, which was in contrast to the decrease in the expression of most of the IAA signaling-related genes. A combined transcriptomics and metabolomics analysis revealed that flavonoids may help regulate plant organ abscission. A weighted gene co-expression network analysis detected two gene modules significantly related to abscission. The genes in these modules were mainly related to exosome, flavonoid biosynthesis, ubiquitin-mediated proteolysis, plant hormone signal transduction, photosynthesis, and cytoskeleton proteins. Furthermore, *TIP1;1*, *UGT71C4*, *KMD3*, *TRFL6*, *REV*, and *FRA1* were identified as the hub genes in these two modules.

**Conclusions:**

In this study, we elucidated the mechanisms underlying cotton boll abscission induced by shading on the basis of comprehensive transcriptomics and metabolomics analyses of the boll abscission process. The study findings have clarified the molecular basis of cotton boll abscission under low light intensity, and suggested that H_2_O_2_, phytohormone, and flavonoid have the potential to affect the shedding process of cotton bolls under low light stress.

**Supplementary Information:**

The online version contains supplementary material available at 10.1186/s12870-024-04862-7.

## Introduction

Cotton (*Gossypium hirsutum* L.) is one of the most important cash crops worldwide because it is the main source of natural fibers in the textile industry. Under natural conditions, there are a variety of factors affecting normal cotton plant growth and development. The shedding of squares and young bolls is a significant factor that substantially limits the cotton yield. Previous research indicated that the square and boll shedding rate is typically approximately 60%–70% [1]. Developing elite breeding materials with decreased boll shedding rates in response to environmental stimuli may be a viable strategy for increasing cotton yields.

The shedding of squares and young bolls involves the formation of an abscission zone (AZ) in the peduncle–fruiting branch junction. The abscission of plant organs is a complex process regulated by genes, enzymes, and hormones. In plants, numerous genes are involved in regulating AZ formation. For example, the LMBP21-J-MC complex encoded by *JOINTLESS*, *MACROCALYX*, and *SLMBP21* helps regulate pedicel AZ development in tomato [2, 3]. Loss-of-function mutations to these genes lead to a lack of flower AZs [4]. The loss of the seed shattering trait (i.e., developmentally programmed abscission) was crucial for the domestication of cereal crops. In rice, the *Shattering Abortion 1* (*OsSHAT1*) gene, which affects the differentiation of the seed abscission layer, is essential for seed shattering [5]. The *OsqSH1* gene encodes a BELL-type homeobox that facilitates the development of the basal abscission layer of the glume, thereby promoting seed abscission [6, 7]. Additionally, *OsSH5*, which is homologous to *OsqSH1*, is highly expressed in the abscission layer of pedicels and may enhance the seed shattering trait [8]. The *SH1* gene encodes a YABBY transcription factor (TF) that contributes to the abscission process in different monocot species, whereas *SpWRKY* encodes a WRKY TF that modulates the wild sorghum seed shattering trait [9]. In addition, some genes involved in cell wall degradation and remodeling, including genes encoding expansins (EXPs), pectinesterases (PEs), pectinlyases (PLs), xyloglucan endotransglucosylase/hydrolases (XTHs), and β-glucosidases (BGLUs), have been identified in sweet cherry, tomato, lychee and other crops, in which they participate in AZ formation [10–12]. Plant hormones are also key contributors to abscission. Earlier research showed that endogenous auxin controls the sensitivity to ethylene, with a decrease in the auxin concentration resulting in increased sensitivity to ethylene, ultimately leading to plant organ shedding [13]. Another study determined that SlPIN1-regulated auxin efflux influences the flower abscission process [14]. To date, several studies on blue honeysuckle, tomato flowers, and fruit trees revealed that considerable changes to plant hormone contents promote the abscission of fruit organs [15, 16].

During the cotton growth period, many factors, such as light intensity, moisture levels, temperature, nutrient availability, insect infestations, and pathogen infections, can affect the shedding of squares or bolls. Some studies have demonstrated that an exposure to low light intensity leads to premature square and boll shedding because of the associated delays in photosynthesis and decreases in the supply of organic nutrients [17, 18]. However, there have been relatively few studies on the effects of low light intensity on the mechanisms regulating abscission. Transcriptomics and metabolomics research techniques are useful for exploring the molecular mechanisms underlying biological processes. Moreover, they may be applied to clarify the changes in gene expression and metabolite contents during the cotton boll abscission process. In this study, we shaded the leaves on fruiting branches with cotton bolls after pollination to simulate low light conditions. We examined the effects of shading on abscission and analyzed the transcriptome and metabolome of the AZ region of young cotton bolls. The objective of this study was to identify the genes and endogenous metabolites involved in AZ formation in cotton bolls under low light intensity.

## Materials and methods

### Plant materials and treatments

The cotton varieties Ji172 used in this study were from Institute of Cotton, Hebei Academy of Agriculture and Forestry Sciences. Cotton plants were grown under field conditions following common agronomic practices. We selected young cotton bolls from the first fruit section of the eleventh fruiting branch as the test material. After pollination, we covered the leaves of this fruiting branch with a brown paper envelope, and in order to further restrict nutrient transport, fruiting branches were girded (S treatment). The AZ tissue (peduncle–fruiting branch junction) of the first fruit boll was collected at 0 day(S0), 1 day (S1), 2 days (S2), and 3 days (S3) after the S treatment. Samples that were left uncovered were collected at the same time-points (W1, W2, and W3)and were used as the controls. All samples were immediately frozen in liquid nitrogen and stored at − 80 °C until analyzed.

### Microscopic examination of the AZ and analysis of the abscission rate

Paraffin sections of the AZ were prepared to further analyze abscission layer formation using the Leica TCS SP8 microscope (LEICA, Italy, Germany). The paraffin sections were prepared as previously described [19] using the Leica RM2125 RTS microtome (LEICA, Italy, Germany).

Shedding was considered to have occurred if samples fell off after being lightly touched with tweezers. The abscission rate (%) was calculated using the following formula: (number of bolls before the treatment − number of bolls after the treatment)/number of bolls before the treatment) × 100.

### Determination of the O_2_^−^ and H_2_O_2_ contents

The O_2_^−^ and H_2_O_2_ contents were determined using the Superoxide Anion kit and the Hydrogen Peroxide kit (Grace Biological Co., Ltd, Suzhou, China). The study data were obtained from more than three independent replicates and were statistically analyzed by Student’s t-test. Standard deviations were calculated for the means and significant differences were determined using *P* < 0.05 and *P* < 0.01 as the thresholds.

### Untargeted metabolomics analysis

Samples were sent to Shanghai Zhongke New Life Biotechnology Co., Ltd. for the untargeted metabolomics analysis. After the chromatographic separation step, the metabolites were analyzed using the AB Triple TOF 6600 mass spectrometer. The raw data were converted to the mzXML format using the ProteoWizard software. The peak alignment, retention time correction, and peak area extraction were performed using the MSDAIL software. Finally, the mass spectrometry data underwent the following bioinformatics analyses: principal component analysis (PCA) [20] and KEGG pathway analysis. All samples were examined using six biological replicates.

### Transcriptomics analysis

Total RNA was extracted from the collected samples and sent to Biomarker Technologies Co., Ltd. (Beijing, China) for the paired-end sequencing using the Illumina NovaSeq 6000 system. The raw sequence data reported in this paper have been deposited in the Genome Sequence Archive (Genomics, Proteomics & Bioinformatics 2021) in National Genomics Data Center (Nucleic Acids Res 2022), China National Center for Bioinformation / Beijing Institute of Genomics, Chinese Academy of Sciences (GSA: CRA014293) that are publicly accessible at https://bigd.big.ac.cn/gsa/browse/CRA014293 [21]. The obtained clean reads were compared with the upland cotton reference genome sequence (G.hirsutum_TM-1_ICR.genomic) using the HISAT2 (version 2.0.1) software [22]. The unsupervised PCA was performed using the prcomp function within R. The differentially expressed genes (DEGs) between two samples were analyzed using the R package DESeq2 [23]. The Gene Ontology (GO) and KEGG enrichment analyses of the DEGs were conducted (corrected *P* < 0.05) using the TBtools software [24]. All analyses were performed using three biological replicates per sample.

A weighted gene co-expression network analysis (WGCNA) was conducted using the R-package WGCNA [25]. The adjacency matrix for the correlation between genes was constructed using a threshold power of 14 and the minModuleSize parameter was set at 200. Several genes with the highest kME (characteristic gene connectivity) values were selected as the key genes in each module [26]. The gene regulatory network was constructed using the Cytoscape software (version 3.9.1) [27].

### Quantitative real-time PCR

Total RNA was extracted from each sample as described above. The MonScript RTIII Super Mix with dsDNase (Two-Step) kit was used for the first-strand cDNA synthesis, after which the quantitative real-time PCR (qRT-PCR) analysis was completed using the MonAmp ChemoHS qPCR Mix (Monad, Suzhou, China). The qRT-PCR primer sequences are provided in Supplementary file Table 1. A *histone* gene was selected as the internal reference gene. The qRT-PCR program was as follows: 95 °C for 10 min; 40 cycles of 95 °C for 15 s and 58 °C for 1 min; final dissociation curve analysis. Relative gene expression levels were calculated using the 2^−ΔΔCt^ method [28]. The qRT-PCR analysis was performed using three biological replicates per sample.

## Results

### Abscission rate and morphological characteristics of the AZ during the S treatment

**Fig. 1:**
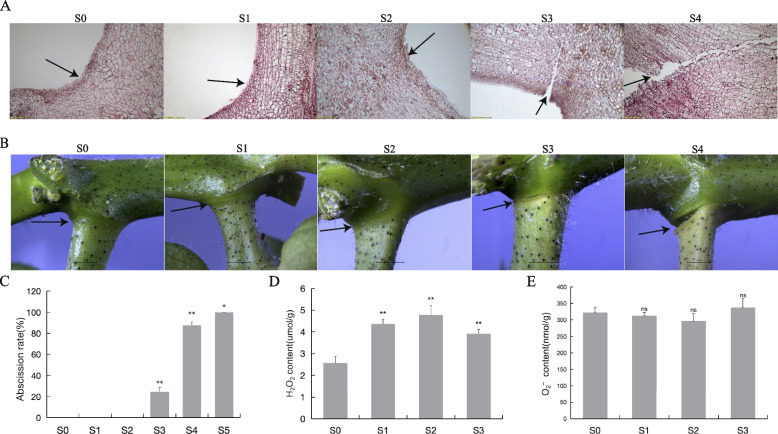
Morphological study of cotton boll shedding process after S treatment and determination of ROS content. AZ: abscission zone. S0-S5 shows treatment 0d, 1d, 2d, 3d, 4d, 5d, respectively. **A**,**B** Paraffin section and Stereoscope to observe effect of S treatment at different times on of abscission zone of cotton boll. **C** Investigation on the shedding rate of cotton bolls at different time points after S treatment. Statistical significance between different treatment and control groups was tested using T test (*p < 0.05; **p < 0.01), biological replicates *n* = 3. **D**,**E** Changes of H2O2 and O_2_.^−^ content in cotton bolls treated with S. Statistical significance between different treatment and control groups was tested using T test (*p < 0.05; **p < 0.01), biological replicates *n* = 6

Cotton boll abscission was examined after the S treatment. There were no abscising young bolls during the first and second days after the S treatment. However, on the third day after the S treatment, the young bolls started abscising, with an abscission rate of 24.4%. On the fourth day after the S treatment, there was a significant increase in the abscission rate, which peaked at 63.3%. By the fifth day after the S treatment, all of the remaining young bolls had abscised (Fig. 1C). To analyze the formation of the abscission layer, the formation process of the abscission layer was observed using a paraffin sections (Fig. 1A) and stereoscope (Fig. 1B). The abscission layer was undetectable during the first two days after the S treatment, but on the third day, an abscission layer was clearly detected. On the fourth day, the abscission layer became more prominent and most cotton bolls dropped when lightly touched using tweezers.

### Reactive oxygen species contents during abscission

Reactive oxygen species (ROS) (e.g., H_2_O_2_, superoxide anion, singlet oxygen, and hydroxyl radical) are generated in response to environmental stress. Previous studies suggested there is a link between ROS and abscission [29, 30]. To assess the effects of ROS on cotton boll abscission, the O_2_^−^ and H_2_O_2_ contents in the AZ after the S treatment were determined. The H_2_O_2_ content increased after the S treatment, reaching peak levels at 2 days post-treatment (Fig. 1D). In contrast, there were no significant changes to the O_2_^−^ content (Fig. 1E). These results reflect the accumulation of H_2_O_2_ in the AZ during the formation of the abscission layer, suggestive of its importance for cotton boll abscission.

### Metabolomics analysis

**Fig. 2:**
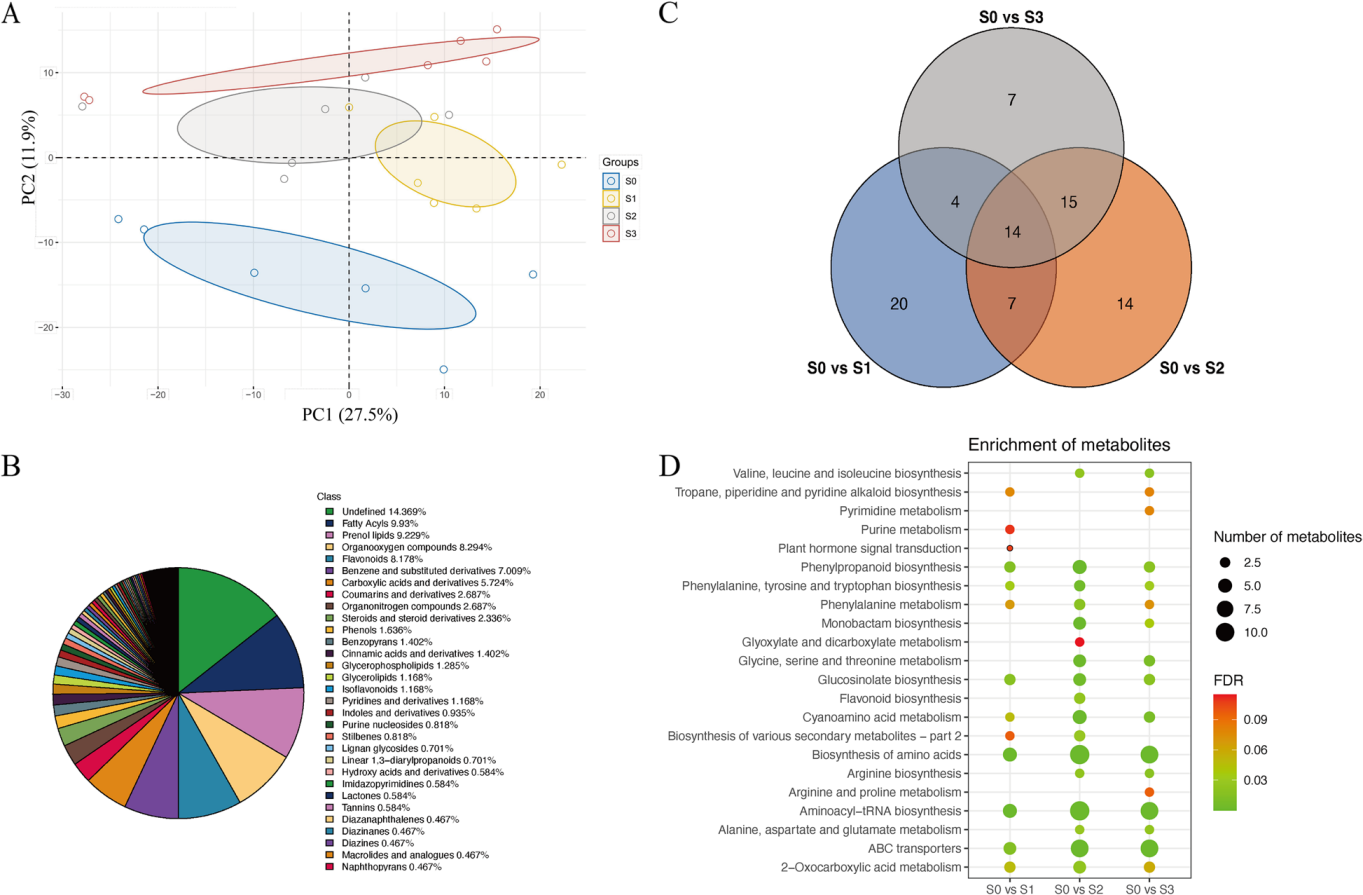
Metabolome analysis of cotton boll shedding process after S treatment. **A** Each point in PCA score plot representing an independent biological replicate. **B** A pie plot showing the classification of all metabolites detected at all time points. **C** A veen diagrams showing differentially accumulated metabolites (DAMs) at different times point. **D** A bubble plot showing KEGG enrichment analysis of DAMs at different time points

To further clarify the physiological and molecular mechanisms underlying cotton boll abscission after the S treatment, 24 samples (six replicates each of S0, S1, S2, and S3) were prepared for the untargeted metabolomics analysis. A total of 856 metabolites were detected. According to the PCA results, the sample groups following the S treatment were clearly separated, indicative of significant changes to the metabolite contents during the formation of the abscission layer after the S treatment (Fig. 2A). The KEGG annotation results identified the main metabolites as fatty acyls, prenol lipids, isopentenols, organooxygen compounds, flavonoids, as well as benzene and substituted derivatives (Fig. 2B). These metabolites may be crucial for cotton boll abscission.

To identify the differentially accumulated metabolites (DAMs) in three boll abscission developmental stages, we performed pair-wise comparisons of the control (S0) and S-treated samples at different time-points using the following criteria: fold-change > 1, VIP ≥ 1, and *P* < 0.05. The three pair-wise comparisons detected 45 DAMs (32 up-regulated and 13 down-regulated), 50 DAMs (27 up-regulated and 23 down-regulated), and 40 DAMs (25 up-regulated and 15 down-regulated) (Fig. 2C). Some of the enriched KEGG pathways among the DAMs were similar to the enriched KEGG pathways among the DEGs, including “Plant hormone signal transduction,” “Flavonoid biosynthesis,” and “Phenylalanine metabolism” (Fig. 2D).

### RNA‑Seq analysis and identifcation

**Fig. 3:**
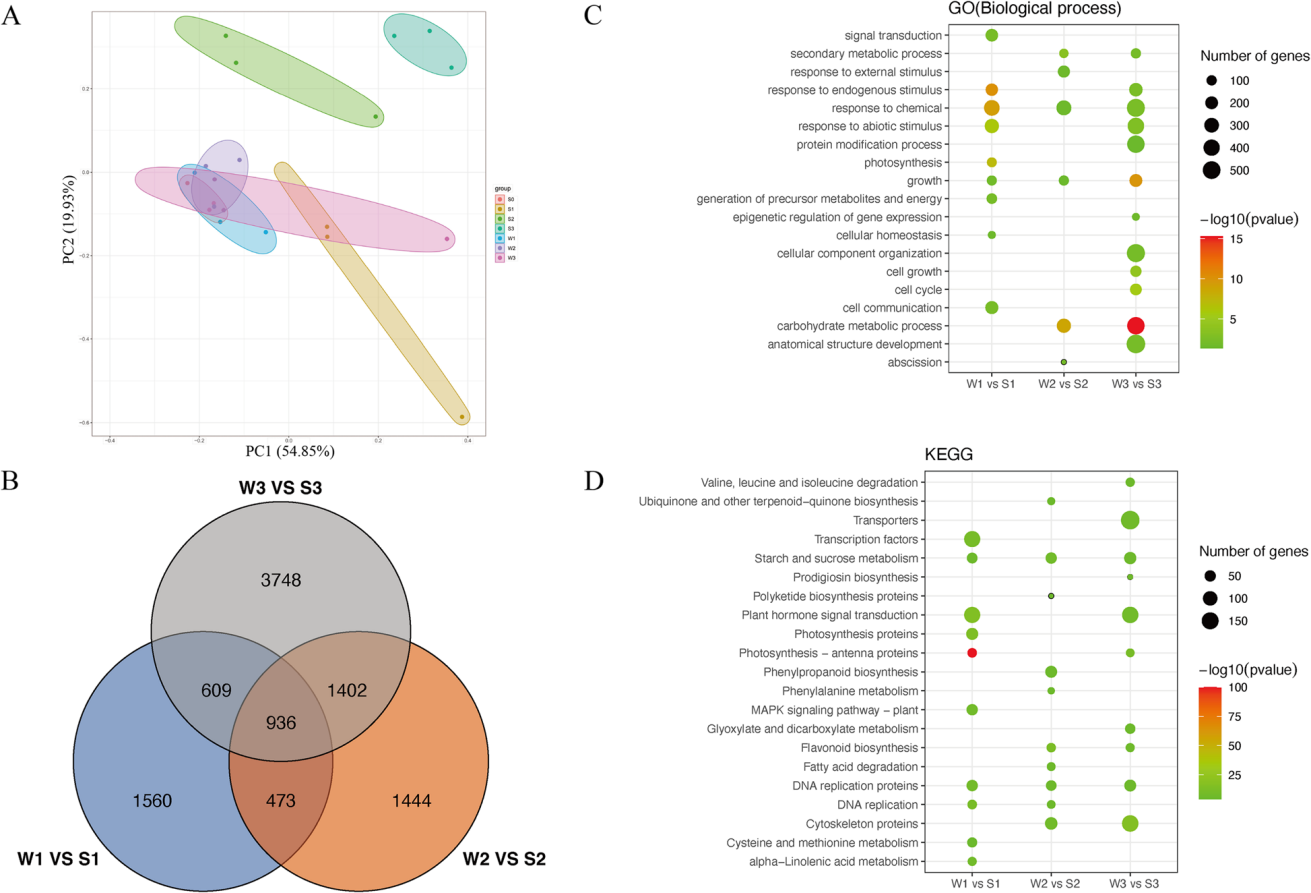
Transcriptome analysis of cotton boll shedding process after S treatment. **A** Each point in PCA score plot representing an independent biological replicate. **B** A veen diagram showing differentially expressed genes (DEGs) at different times point. **C**,**D** GO and KEGG enrichment analysis of DEGs at different time points

To explore the molecular basis of cotton boll abscission under low light intensity, 21 samples were collected at four time-points (0, 1, 2, and 3 days) with and without the S treatment for the RNA-seq analysis. After removing the low-quality data, 28,589,054–19,119,870 clean reads were retained for each library, with a Q30 exceeding 92.13%. Of these reads, 88.5%–93.5% were mapped to the *G. hirsutum* reference genome sequence (Supplementary Table 2). The PCA results revealed W1, W2, W3, and S0 (untreated group) were clustered together, separate from the S-treated group (Fig. 3A). Hence, there were relatively few differences among the untreated samples, but the S treatment resulted in a significant change in the transcriptome. The pair-wise comparisons of the control samples (W1, W2, and W3) and S-treated samples (S1, S2, and S3) detected 10,172 differentially regulated genes. Specifically, 3,578, 4,255, and 6,695 genes were differentially expressed on days 1, 2, and 3 of the S treatment, respectively, among which 936 were consistently differentially expressed, including 276 up-regulated DEGs and 649 down-regulated DEGs (Fig. 3B). To verify the reliability of the transcriptomics data, we randomly selected 10 DEGs for the qRT-PCR analysis. The results showed there was a strong correlation between the qRT-PCR and RNA-seq results (*R* = 0.95), indicative of the reliability of the transcriptome sequencing data (Supplementary Fig. 1).

### GO and KEGG enrichment analyses of the DEGs following the S treatment

The DEGs were functionally annotated on the basis of GO and KEGG enrichment analyses. “Response to chemical” and “growth” were the common enriched GO terms at all S treatment time-points. Five GO terms (“signal transduction,” “photosynthesis,” “generation of precursor metabolites and energy,” “cellular homeostasis,” and “cell communication”) were enriched only among the DEGs on day 1 of the S treatment, whereas “abscission” was an enriched GO term exclusive to the DEGs on day 2 of the S treatment. The DEGs on day 3 of the S treatment were mainly annotated with the following GO terms: “secondary metabolic process,” “response to endogenous stimulus,” “response to abiotic stimulus,” “protein modification process,” “epigenetic regulation of gene expression,” “cellular component organization,” “cell growth,” “cell cycle,” and “anatomical structure development” (Fig. 3C).

The KEGG enrichment analysis indicated “Starch and sucrose metabolism” and “DNA replication proteins” were the common enriched pathways at all S treatment time-points. The main enriched KEGG pathways among the DEGs on day 1 of the S treatment were “Transcription factors,” “Plant hormone signal transduction,” “Photosynthesis proteins,” “Photosynthesis – antenna proteins,” and “MAPK signaling pathway – plant.” Notably, the DEGs associated with the plant hormone signal transduction pathway mainly included genes related to ABA, IAA, and ETH signal transduction. Moreover, the expression levels of all of the ETH-related genes were up-regulated, suggesting ethylene plays an important role in the early abscission layer formation stage following the S treatment. The “Flavonoid biosynthesis” and “Cytoskeleton proteins” KEGG pathways were significantly enriched among the DEGs on days 2 and 3 of the S treatment (Fig. 3D).

### Time-course analysis of DEGs expression

**Fig. 4:**
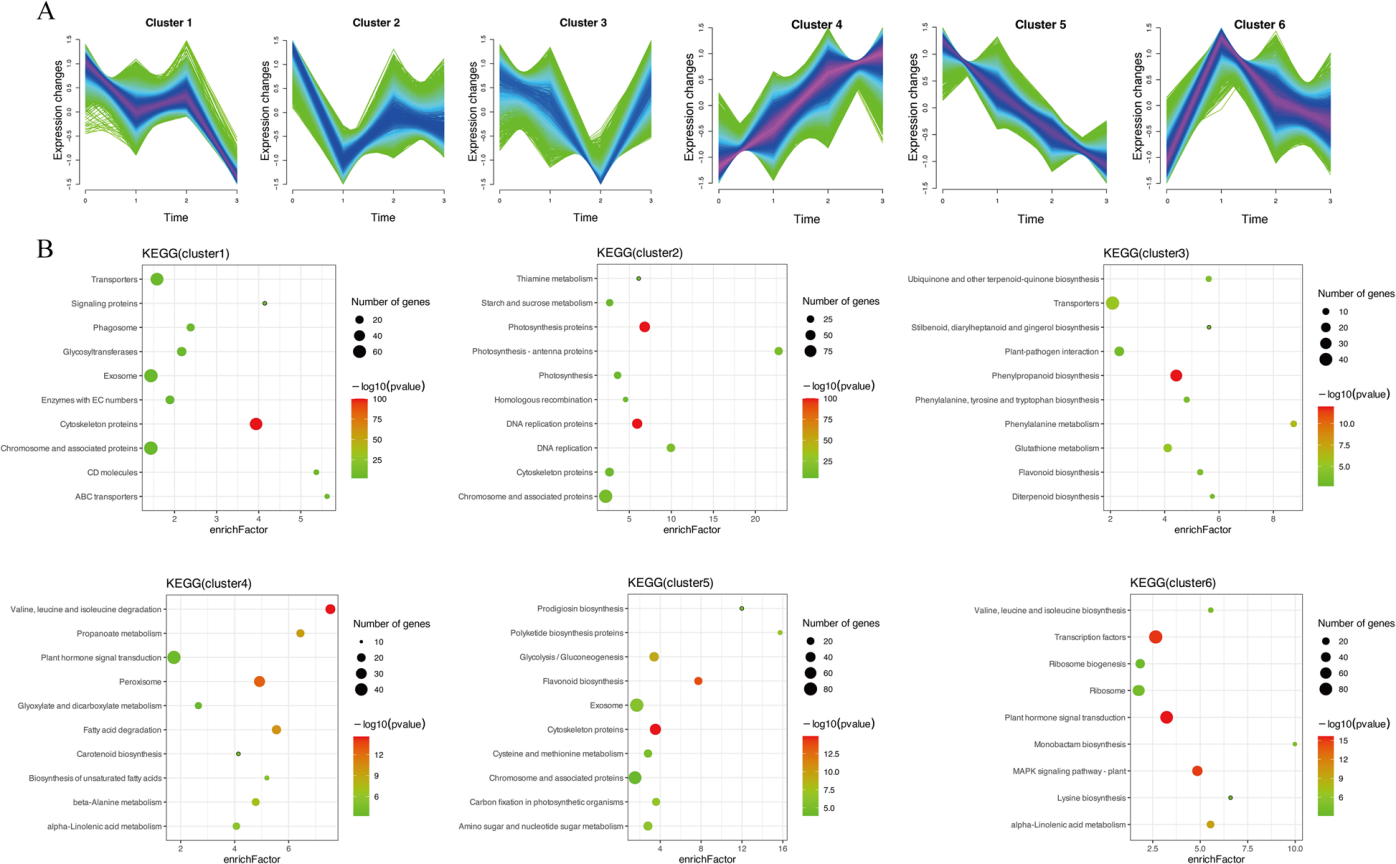
K-means cluster analysis of DEGs and KEGG enrichment analysis of each cluster. **A** K-means cluster analysis of DEGs. **B** KEGG enrichment analysis of each cluster

To examine the DEG spatiotemporal expression patterns, a time-course analysis of gene expression was performed using the Mfuzz package. Genes with similar expression patterns may be involved in the same biological processes. The DEGs identified in this study were classified into six clusters (Fig. 4A). The main enriched KEGG pathways in each cluster are shown in the Fig. 4B. The expression levels of the genes in clusters 1 and 2 tended to decrease over time. Additionally, the main enriched pathways differed between the cluster 1 genes (“Cytoskeleton proteins” and “Transporters”) and the cluster 2 genes (“DNA replication proteins” and “Photosynthesis proteins”). The expression levels of the cluster 3 genes initially decreased, but then gradually increased. The enriched pathways among these genes were “Phenylpropanoid biosynthesis” and “Transporters.” The cluster 4 genes had continuously increasing expression levels and were primarily involved in degradation-related pathways (e.g., “Valine, leucine and isoleucine degradation” and “Fatty acid degradation”). Thus, shade stress may trigger the degradation of proteins and other substances, resulting in the formation of the abscission layer. “Peroxisome” and “Plant hormone signal transduction” were also significantly enriched pathways in cluster 4. The cluster 5 genes had gradually decreasing expression levels. The enriched pathways among these genes were “Cytoskeleton proteins,” and “Flavonoid biosynthesis.” On the basis of the enriched pathways (e.g., “Plant hormone signal transduction,” “MAPK signaling pathway,” and “Transcription factors”), the genes in cluster 6 were associated with several biological processes involving signal transduction. Moreover, the cluster 6 gene expression levels increased rapidly after the S treatment, suggesting these genes may affect the early response to shading stress.

### DEGs involved in plant hormone signal transduction pathways

**Fig. 5:**
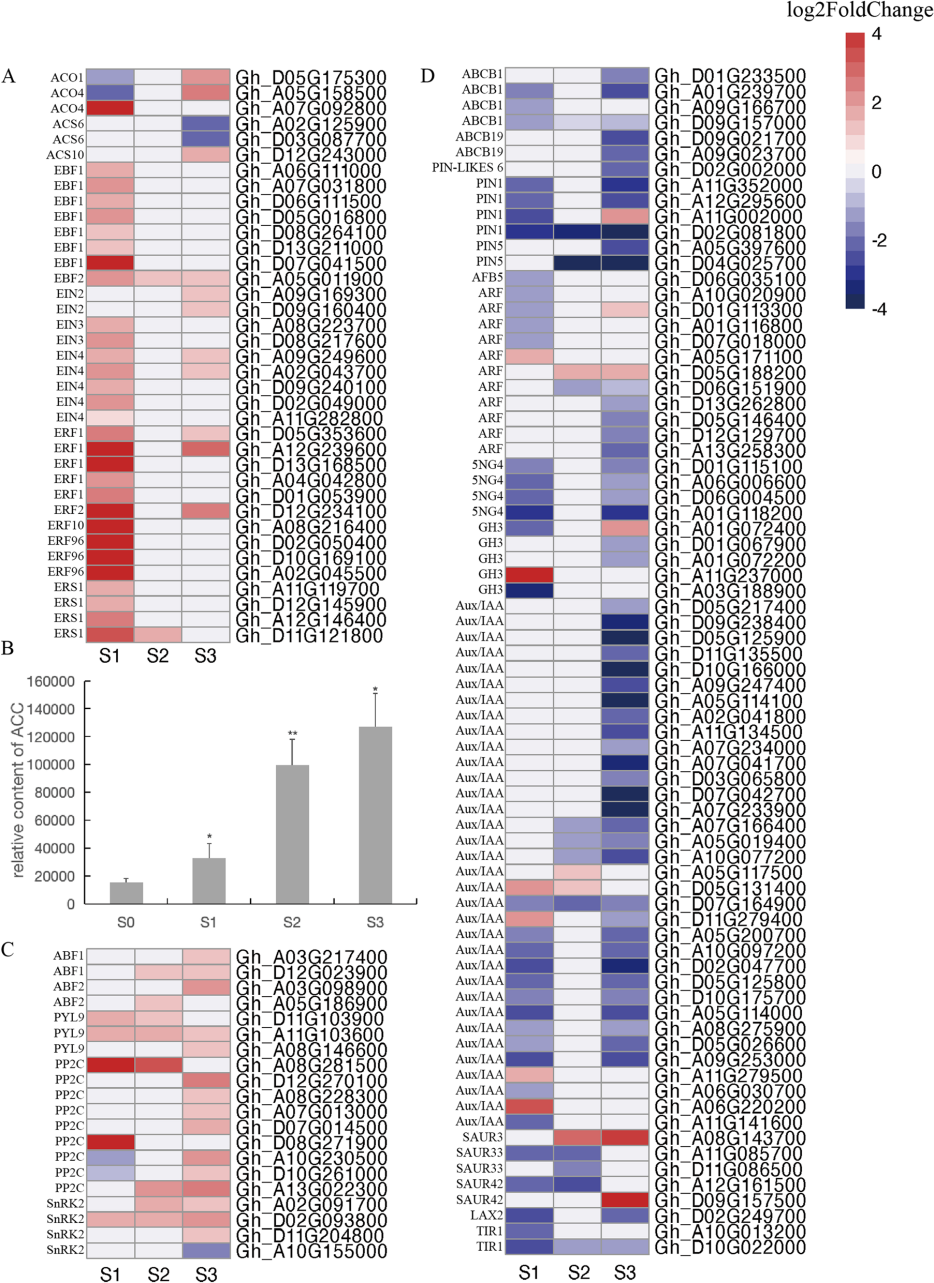
Heat maps of DEGs involved in the biosynthesis and signal transduction of ethylene, abscisic acid and auxin. Gene expression levels are indicated with color bars and red represents up-regulated expression and blue represents down-regulated expression. Histogram showing the change of 1-aminocyclopropane-1-carboxylic acid (ACC) content at AZ of cotton boll. **A** Analysis of gene expression trends in ethylene synthesis and signal transduction pathways. **B** The change trend of 1-aminocyclopropane-1-carboxylic acid (ACC). Statistical significance between different treatment and control groups was tested using T test (*p < 0.05; **p < 0.01), biological replicates *n* = 6. **C** Analysis of gene expression trends in abscisic acid synthesis and signal transduction pathways. **D** Analysis of gene expression trends in auxin synthesis and signal transduction pathways

A previous study demonstrated the importance of plant hormones for abscission [31]. On the basis of our KEGG enrichment analysis, the DEGs involved in plant hormone signal transduction pathways mainly included genes related to ABA, IAA, and ETH signal transduction. Specifically, 37 genes were associated with the ethylene signal transduction pathway. Among these genes, most of the ethylene synthesis-related genes (e.g., *ACS* and *ACO*) and ethylene receptor-encoding genes (*EIN4* and *ERS1*) had up-regulated expression levels (Fig. 5A). The metabolomics analysis detected a significant increase in the 1-aminocyclopropane-1-carboxylate (ACC) content (i.e., ethylene precursor) soon after the S treatment was initiated, with peak levels on day 3 (Fig. 5B). The expression levels of most of the genes encoding EIN2, EIN3, EBF, and ERF were significantly up-regulated on day 1 of the S treatment, indicating that ethylene signal response-related genes play an important role during the early abscission layer formation stage under shaded conditions(Fig. 5A).

A total of 20 DEGs were associated with the ABA signal transduction pathway, including nine PP2C genes, four SnRK2 genes, three PYL genes, and four ABF genes. The expression levels of *Gh_A10G230500* and *Gh_D10G261000*, which encode PP2C, initially decreased and then increased, whereas the expression of *Gh_A10G155000*, which encodes SnRK2, was consistently down-regulated. Conversely, the PYL and ABF genes as well as the other SnRK2 and PP2C genes had up-regulated expression levels (Fig. 5C). In addition, there were 76 DEGs encoding diverse proteins (e.g., Aux/IAA, ARF, 5NG, LAX, PIN, PIN-LIKES, CH3, AFB, TIR, SAUR, and ABCB) associated with the IAA signal transduction pathway. The majority of the IAA signal transduction-related genes had down-regulated expression levels (Fig. 5D).

### Expression of TF genes

**Fig. 6:**
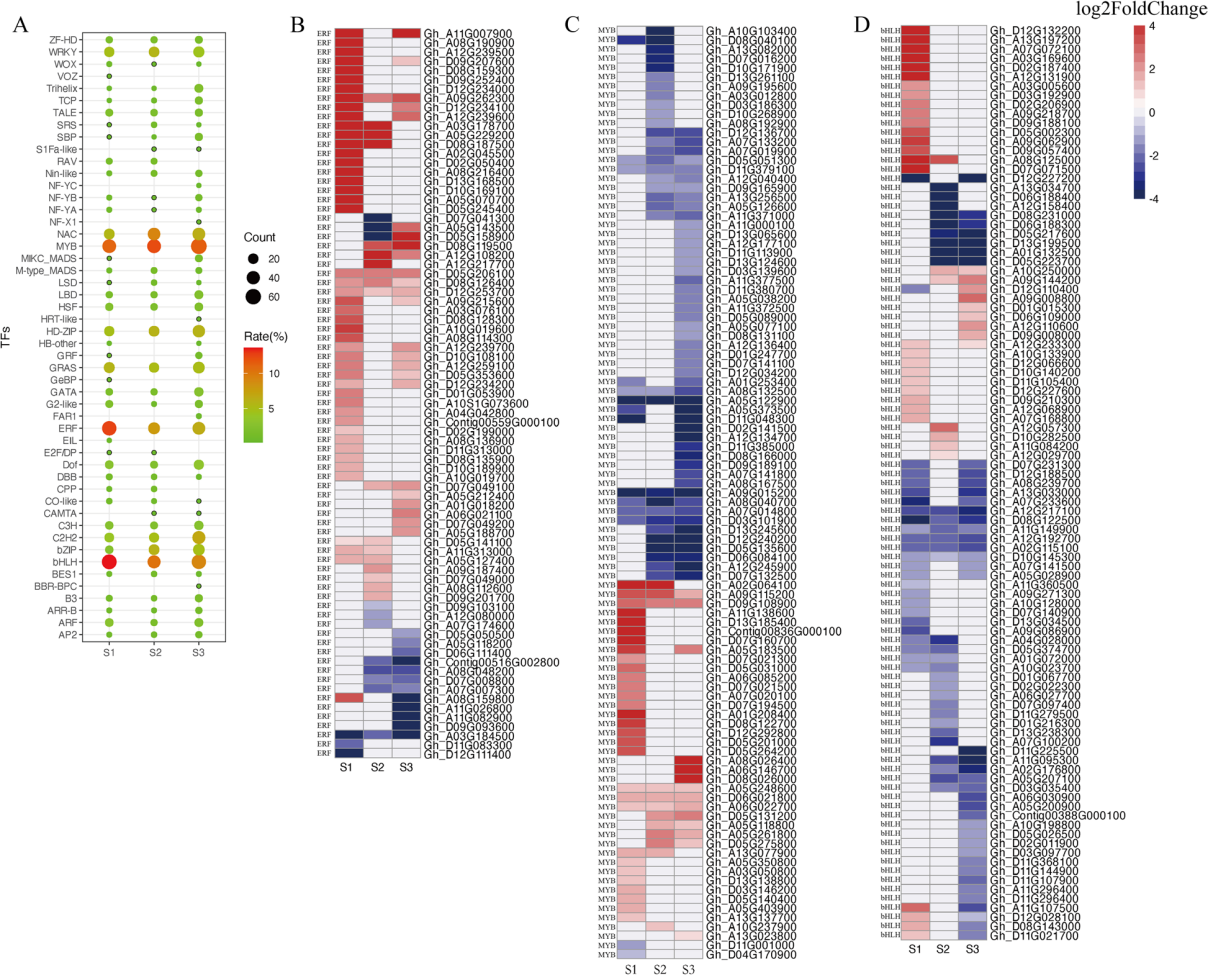
Bubble plot of all transcription factors detected in AZ and heat maps of ERF, MYB and bHLH family transcription factors. Gene expression levels are indicated with color bars and red represents up-regulated expression and blue represents down-regulated expression. **A** A bubble map showing transcription factors belonging to 50 families. **B** Expression trend analysis of ERF family transcription factors. **C** Expression trend analysis of MYB family transcription factors. (D) Expression trend analysis of bHLH family transcription factors

Transcription factors are essential regulators of the cellular changes during AZ formation in response to developmental and environmental signals. In this study, 914 differentially expressed TF genes belonging to 50 families were identified using the default parameters of PlantTFDB 5.0. The ERF, MYB, and bHLH family members were the predominant TFs (Fig. 6A). The expression levels of most of the *ERF* genes were significantly up-regulated on day 1 of the S treatment (Fig. 6B). Of the genes encoding MYB and bHLH TFs, approximately one-third had up-regulated expression levels, whereas the rest had down-regulated expression levels (Fig. 6C, D). These TF genes included three homologous genes that regulate abscission. Specifically, *Gh_A05G087000* and *Gh_D05G098700* encode AP2 and are homologous to *SSH1*. In rice, SSH1 positively regulates the expression of two shattering-related genes (*qSH1* and *SH5*), thereby affecting lignin deposition in the AZ and abscission layer development, which influences seed shattering [32]. The *Gh_A01G208400* gene, which encodes a MYB TF, is homologous to *SvLes1*, which has been associated with seed shattering in *Setaria viridis* [33]. Whether these three genes modulate cotton boll abscission remains to be determined.

### Genes related to cell wall biosynthesis and degradation

**Fig. 7:**
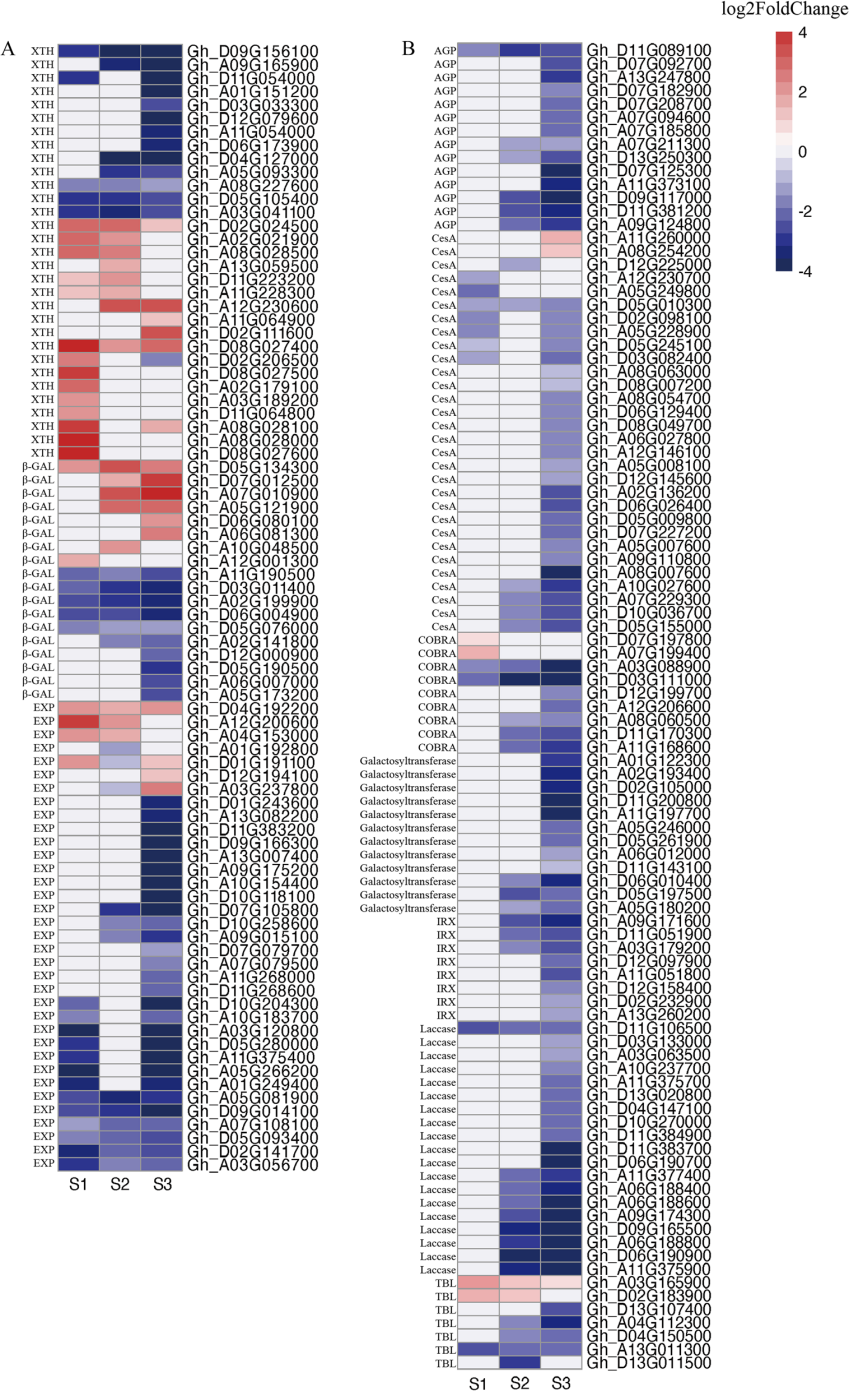
Heat maps of genes associated with cell wall degradation and cell wall synthesis. Gene expression levels are indicated with color bars and red represents up-regulated expression and blue represents down-regulated expression. **A** Analysis of gene expression trends related to cell wall degradation. **B** Analysis of gene expression trends related to cell wall synthesis

Cell wall-remodeling enzymes play crucial roles during abscission. A total of 135 genes associated with cell wall degradation were identified, including genes encoding XTHs, β-galactosidase (β-GAL), BGLUs, EXPs, PEs, and PLs. The cell wall-loosening enzyme XTH hydrolyzes and extends the existing cell wall. Of the 31 genes encoding XTH family members, 18 were DEGs with up-regulated expression levels. Expansins help loosen cell walls by disrupting the bond between cellulose and glycans. In this study, 35 DEGs were identified as EXP family members, of which six genes had up-regulated expression levels. Earlier research indicated β-GAL has a key role affecting abscission as well as early flower and fruitlet growth and development [34], of the β-GAL-encoding genes identified in the current study, eight had up-regulated expression levels (Fig. 7A).

We also identified 99 cell wall-related genes, including genes encoding CesA, laccase, AGP, COBRA, and IRX. The expression of most of these genes was down-regulated (Fig. 7B). In addition, 93 down-regulated genes were associated with cell wall degradation and loosening, whereas six up-regulated genes were involved in cell wall biosynthesis. Accordingly, these genes may mediate cell wall synthesis and reconstruction, resulting in the formation of a protective layer following the abscission of bolls.

### Combined transcriptomics and metabolomics analysis of the flavonoid synthesis pathway

**Fig. 8:**
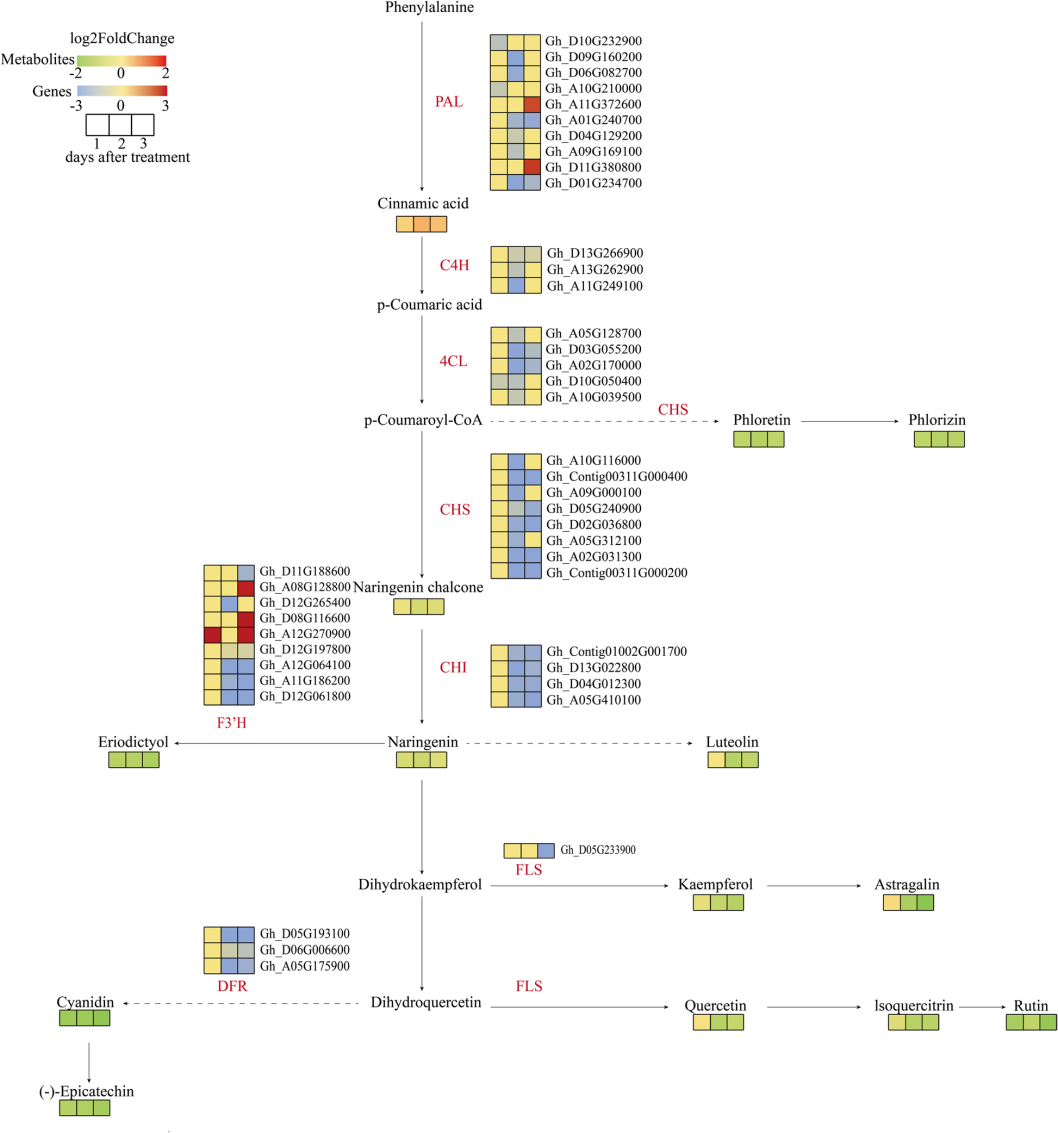
Different expressions of structural genes and metabolites in flavonoid biosynthesis pathway. Red block represent up-regulation and green, blue represent downregulation of expression, respectively. Enzyme annotation: PAL (Phenylalanine ammonia lyase), C4H (Cinnamate4-hydroxylase), 4CL (4—coumarate:CoA ligase), CHS (Chalcone synthase), CHI (Chalcone isomerase), F3’H (Flavonoid 3-hydroxylase), FLS (Flavonol synthase), DFR (Dihydroflavonol 4-reductase)

In this study, DEGs and DAMs significantly related to flavonoid synthesis were identified. The subsequent analysis of these DEGs and DAMs confirmed that 105 DEGs and 70 related metabolites were associated with flavonoid production. The examination of the DEGs and metabolites associated with flavonoid synthesis-related metabolic pathways indicated that the expression of most of the genes related to flavonoid formation were down-regulated, which was consistent with the decrease in the abundance of the related metabolites (Fig. 8), suggestive of the negative effects of flavonoids on abscission.

### Metabolic and gene co-expression networks in different boll abscission developmental stages

**Fig. 9:**
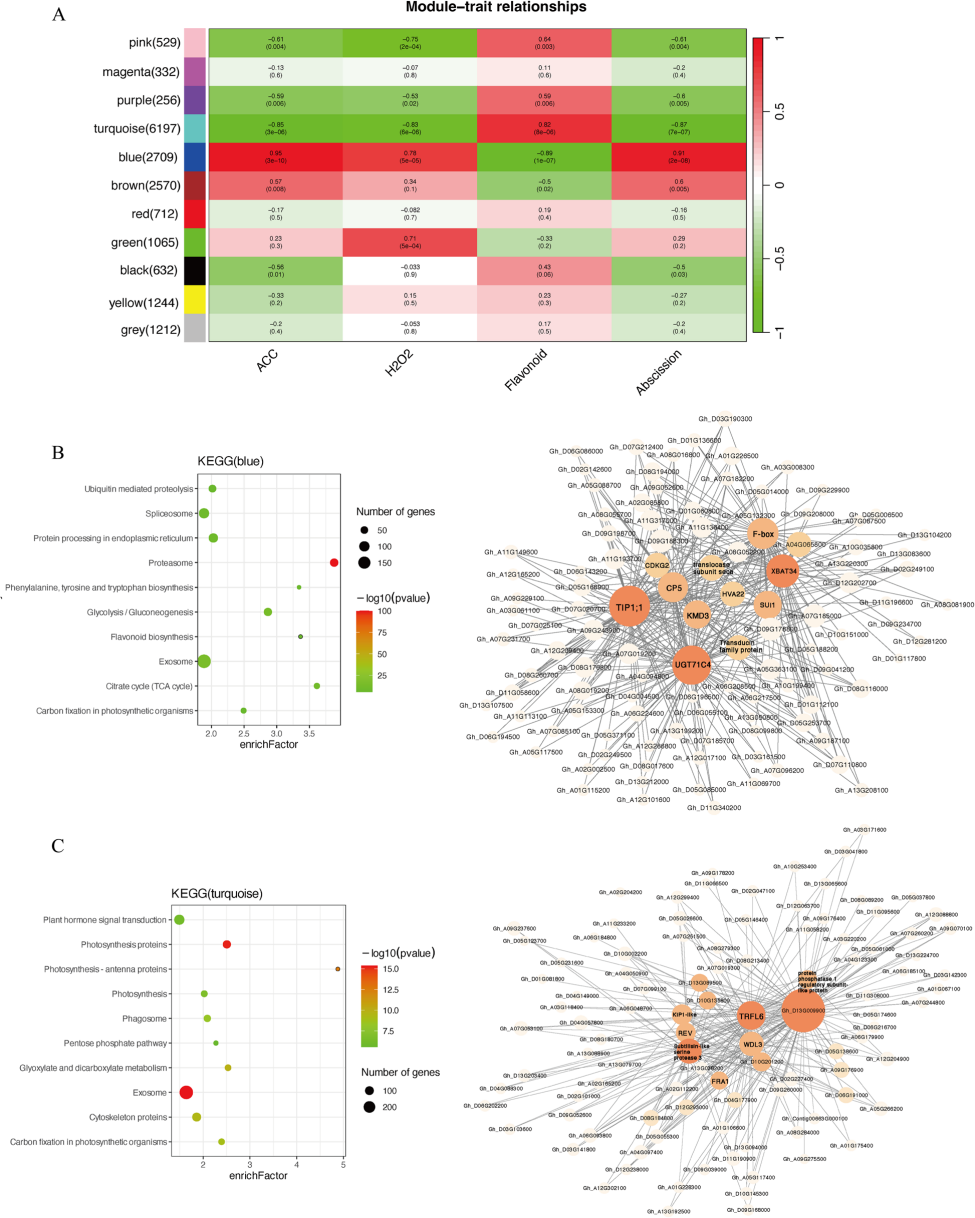
Identification of key modules and hub genes associated with the shedding of cotton bolls by WGCNA. **A** Heat map of the correlation between modules and phenotypes: each row corresponds to a ME and each column corresponds to a phenotype. The data in the cells represent the corresponding correlation and *P*-value. The red key represents a positive correlation between modules and phenotypes, while the green key represents the opposite. **B** KEGG enrichment pathway analysis of blue module genes and visualization of the hub genes co-expression network of the blue module. **C** KEGG enrichment pathway analysis of turquoise module genes and visualization of the hub genes co-expression network of the turquoise module

A WGCNA was conducted to identify co-expressed gene modules and explore the associations between gene networks and metabolite contents during abscission. The most variable genes (top 30%) according to the variance analysis were screened to construct a co-expression network. A total of 11 modules were obtained for the scale-free network. Among these modules, the blue module was highly positively correlated with ACC, H_2_O_2_, and abscission, but highly negatively correlated with flavonoids. The turquoise module was negatively correlated with ACC, H_2_O_2_, and abscission, but highly positively correlated with flavonoids (Fig. 9A).

To functionally annotate the genes in these two modules, a KEGG enrichment analysis was performed. The main enriched pathways among the genes in the blue module were “ubiquitin-mediated proteolysis,” “Spliceosome,” “Proteasome,” “Flavonoid biosynthesis,” and “Exosome” (Fig. 9B). The significantly enriched pathways among the genes in the turquoise module were “Plant hormone signal transduction,” “Photosynthesis proteins,” “Exosome,” and “Cytoskeleton proteins” (Fig. 9C).

To further explore the key genes related to abscission, the genes with the 20 highest kME scores in each module were designated as candidate hub genes (Supplementary Table 3). In the blue module, *GhD04G110100* was annotated as a gene encoding the F-box protein KMD3, which regulates phenylpropanoid biosynthesis by mediating the ubiquitination and subsequent degradation of PAL in *Arabidopsis thaliana *[35]. The *GhA11G371700* gene was revealed to encode a TIP1;1 protein. In tomato, SlTIP1;1 mediates pedicel abscission through its effects on the cytoplasmic H_2_O_2_ concentration and osmotic water permeability [36]. Thus, *GhA11G371700* may be important for cotton boll abscission. In the turquoise module, The *Gh_D05G206400* gene encodes a FRA1 protein which a kinesin-like protein with an N-terminal microtubule binding motor domain, mutants in the gene exhibit altered orientation of cellulose microfibrils and reduced mechanical strength of fibers [37]. *Gh_A08G225500* encodes a REV protein, some studies have reported that BLH6-KNAT7 negatively regulates plant secondary cell wall synthesis, and REV is the direct target of BLH6 and KNAT7, and BLH6 and KNAT7 inhibit secondary cell wall synthesis by inhibiting REV expression [38]. Their potential contributions to abscission are unknown. However, these genes may regulate the shedding process by participating in cell wall metabolism.

## Discussion

Cotton boll abscission is a prevalent problem during cotton production, with considerable effects on the final cotton yield. Cotton boll abscission typically occurs at the young boll stage [39]. A thorough characterization of the mechanisms underlying young boll abscission is critical for improving cotton breeding and cultivation practices.

### Regulatory effects of ROS on boll abscission under low light intensity

Boll abscission often occurs in response to oxidative stresses caused by external factors, including high temperatures, waterlogged or drought conditions, and low light intensity [40]. The accumulation of ROS, such as the superoxide anion (O_2_^−^), hydrogen peroxide (H_2_O_2_), and the hydroxyl radical (OH^−^), is considered to be one of the earliest plant cell responses to external stresses, ultimately leading to oxidative stress. There is increasing evidence of the importance of ROS during the regulation of organ abscission. More specifically, ROS can promote organ abscission, whereas ROS scavengers have the opposite effect. Many studies showed that ROS accumulate in the AZ during organ abscission. For example, a thidiazuron treatment reportedly causes ROS to accumulate in the cotton leaf AZ [41]. In longan, an ROS burst in the pedicel occurs before fruits drop [42]. Among the various ROS, H_2_O_2_ significantly promotes organ abscission. Sakamoto et al. (2008) determined that pepper leaf abscission is enhanced by the application of exogenous H_2_O_2_, whereas it is inhibited by H_2_O_2_ biosynthesis inhibitors or scavengers [43].Cohen et al. (2014) determined that ROS may be responsible for the rapid root abscission in *Azolla* plants*.* Strong H_2_O_2_ signals have been detected in the cytoplasm during abscission [44]. Other studies confirmed H_2_O_2_ is an effective inducer of ETH production [45, 46]. In pepper (*Capsicum annuum*) flowers exposed to osmotic stress, ETH production reportedly increases, ultimately leading to accelerated abscission. However, the introduction of ROS scavengers decreases ETH production and delays abscission [47]. In the present study, the H_2_O_2_ content increased substantially during the formation of the abscission layer after the S treatment, whereas the O_2_^−^ content was relatively unchanged. Additionally, one of the hub genes identified on the basis of the WGCNA was revealed to encode a TIP.1 protein. In tomato, the overexpression of *SlTIP1;1* results in significant increases in apoplastic and cytoplasmic H_2_O_2_ levels. The silencing of *SlTIP1;1* leads to delayed abscission, while the overexpression of this gene accelerates abscission [36]. Therefore, we speculated that cotton boll abscission induced by low light intensity is triggered by H_2_O_2_.

### Hormonal regulation of boll abscission under low light intensity

Previous research indicated that plant hormones, especially ethylene, play important roles during the abscission process [31]. In an earlier study, the application of exogenous ETH caused all litchi fruitlets to drop within 4 days of the treatment [48]. The formation of the abscission layer is promoted by ethylene in both mango [49] and cotton [50]. In accordance with these findings, in another study, the abscission layer started to form at 12 h after a treatment with 400 mg/L ethylene treatment of cotton bolls [51]. However, some studies have shown that although ethylene can accelerate abscission under many conditions, it is not essential [52]. Ethylene synthesis involves the conversion of S-adenosylmethionine to ACC in a reaction catalyzed by ACC synthase (ACS). The ACC is subsequently oxidized by ACC oxidase (ACO), resulting in the production of ethylene, carbon dioxide, and cyanide [53]. Notably, ACS (rate-limiting enzyme) and ACO (enzyme that catalyzes the final step in the ethylene biosynthesis pathway) are two critical enzymes for controlling the ethylene production rate. In this study, three ACS-encoding genes were among the DEGs (two down-regulated genes and one up-regulated gene). In contrast, three identified ACO-encoding DEGs had significantly up-regulated expression levels. Our metabolomics analysis detected a significant increase in the ACC content. We also detected the up-regulated expression of ethylene-responsive genes, including genes encoding ethylene response factor (ERF), ethylene insensitive (EIN), and ethylene receptor (ERS) proteins. These results suggest ethylene positively regulates organ abscission under shaded conditions.

Abscisic acid and auxin are also involved in plant organ abscission. More specifically, ABA affects organ abscission indirectly by inhibiting auxin transport or stimulating ethylene production [54], whereas auxin can inactivate AZ cells and make them insensitive to ethylene signals, thereby inhibiting abscission [55–59]. The PP2C ABI4 can inhibit the expression of the IAA transporter gene *PIN1* [60]. The expression of the ABA receptor gene *PYL* can lead to the up-regulated expression of the IAA-responsive *ARF* genes via MYB TFs [61]. Our data indicated that the expression of ABA signaling-related genes, such as *PP2C*, *SnRK2*, and *PYL*, was activated, but the expression of most of the IAA signaling-related genes, including *ARF*, *Aux/IAA*, *PIN*, and *5NG*, was inhibited. These observations are consistent with the findings of earlier investigations that determined ethylene and ABA are the major signals accelerating abscission [59], while auxin has inhibitory effects on abscission [55–59]. Although the precise mechanism through which hormones regulate organ abscission remains unclear, ETH, ABA, and IAA interact to influence cotton boll abscission.

### Flavonoids may help regulate cotton boll abscission

Flavonoids are a group of important plant secondary metabolites that influence many physiological processes, including plant growth, development, and stress responses. Unfortunately, there is insufficient evidence of a direct relationship between flavonoids and abscission layer formation. However, numerous studies have detected significant changes in the expression of flavonoid synthesis-related genes during the formation of the abscission layer. Earlier analyses of the blue honeysuckle transcriptome and metabolome showed flavonoid biosynthesis and phenylpropanoid biosynthesis pathways are induced during fruit abscission under natural conditions [13]. Moreover, there are substantial changes in flavonoid biosynthesis and flavone/flavonol biosynthesis during the calyx abscission process of *Pyrus sinkiangensis* Yu [62]. The RNA-seq and metabolomics data generated in an earlier study on apple fruit abscission under cold conditions revealed significant differences in flavonoid or amino acid concentrations between the “abscission” group and the “normal” group [63]. In the present study, we identified DEGs and DAMs significantly associated with the flavonoid synthesis pathway. These results suggest flavonoids may have regulatory effects on plant organ abscission.

Flavonoids reportedly determine the auxin gradient at the cell and tissue levels by influencing auxin catabolism [64]. In apple, the flavonoid quercetin, which restricts polar auxin transport, significantly inhibits petiole abscission [65]. Exposing tobacco roots to low concentrations of epicatechin and quercetin can increase the free IAA content, but treatments with high epicatechin and quercetin concentrations have the opposite effect, suggesting high flavonoid contents in tobacco seedlings are not conducive to the basipetal transport of free IAA [66]. According to the metabolomics analysis conducted in the current study, the quercetin content tended to decrease during the cotton boll abscission process. Although IAA inhibits abscission, whether flavonoids modulate tissue abscission by influencing IAA transport remains to be investigated. Because flavonoids are important antioxidants that scavenge ROS, they may regulate abscission layer formation by controlling ROS levels, but this possibility will need to be experimentally verified.

## Conclusion

In this study, we analyzed the transcriptome and metabolome profiles of the AZ of cotton bolls under shaded conditions. During AZ formation, the H_2_O_2_ content increased and the expression of ETH and ABA signaling pathway-related genes increased, whereas the expression of the IAA signaling pathway-related genes decreased. Furthermore, the genes associated with cellulose synthesis had down-regulated expression levels, which was in contrast to the up-regulated expression of the genes involved in plant cell wall degradation. Interestingly, the S treatment also affected the flavonoid synthesis pathway, resulting in a decrease in the flavonoid content, indicative of the regulatory effects of flavonoids on plant organ abscission.

**Fig. 10:**
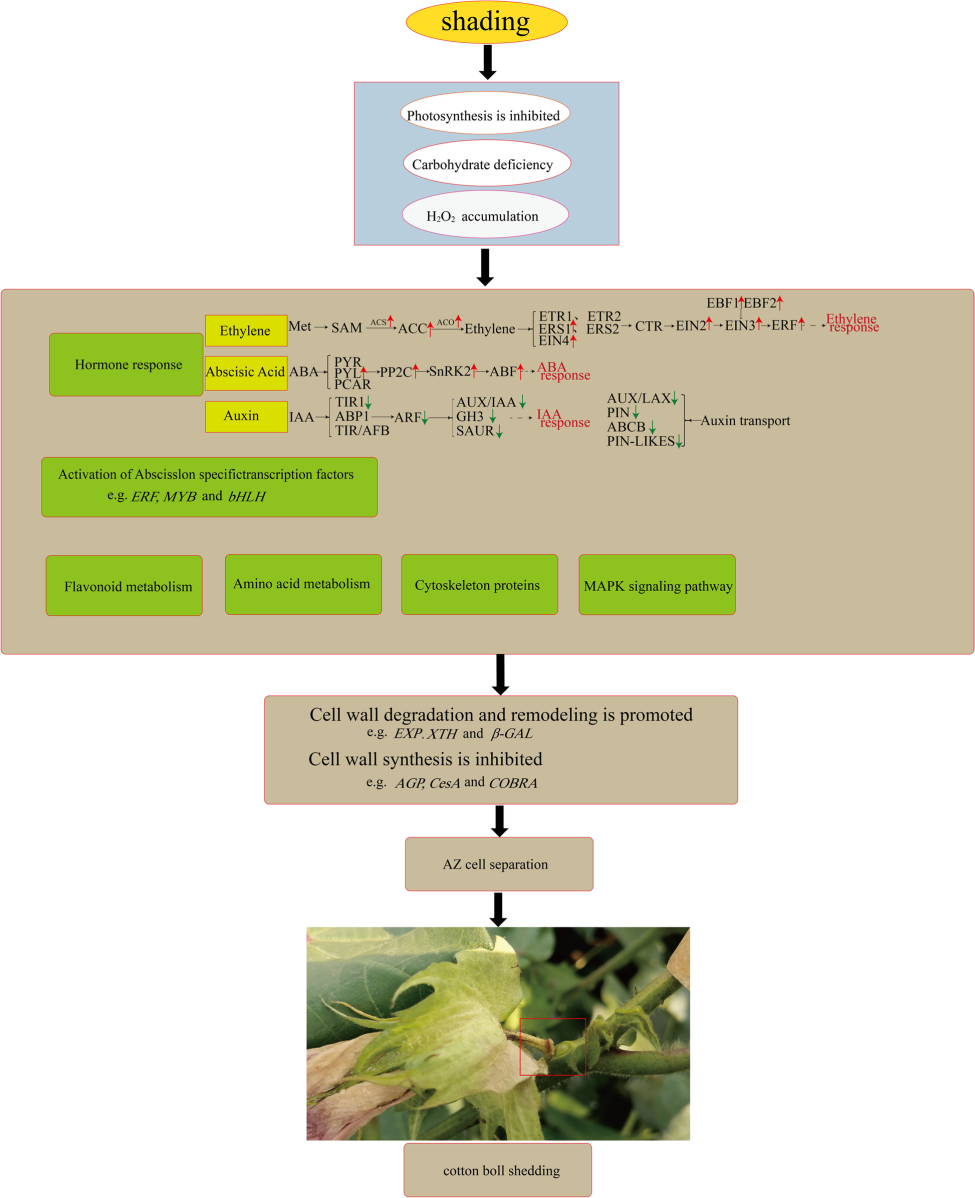
Based on physiological, transcriptome and metabolome data, the possible molecular regulatory events of low light promoting cotton boll shedding were analyzed. The dotted line represents an indirect effect. The red and green arrows indicate the Genes or metabolites involved in plant hormone synthesis and signal transduction pathways up- or down- regulated, respectively

On the basis of the study results, we proposed a gene network that explains the abscission of cotton bolls under shaded conditions (Fig. 10). Briefly, shading results in inhibited photosynthesis, which leads to carbohydrate deficiency and ROS accumulation. Increases in the ROS content can trigger hormone signaling pathways, including ethylene, auxin, and ABA pathways, while also activating certain TFs, such as AP2, ERF, and bHLH family members. Other metabolic pathways may also be involved in this process (e.g., pathways affecting cytoskeletal integrity, amino acid metabolism, MAPK signaling, and flavonoid metabolism). This series of events leads to proteolysis, cell detachment, and cell death, ultimately resulting in cotton boll abscission.

### Supplementary Information

**Additional file 1: Table S1.** Primer sequences.

** Additional file 2: Table S2.** Transcriptome sequencing data.

** Additional file 3: Table S3.** Hubgene and its annotation in blue and turquoise modules.

** Additional file 4: Fig. S1.** qRT-PCR analysis of 10 randomly selected genes.

## Data Availability

The data presented in the study are deposited in the NGDC repository, accession number PRJCA022626.

## Abbreviations

ACC: 1-Aminocyclopropane-1-carboxylate
ACO: 1-Aminocyclopropane-1-carboxylate oxidase
AZ: Abscission zone
BGLU: β-Glucosidases
DAM: Differentially accumulated metabolite
ERF: Ethylene response factor
EIN: Ethylene insensitive
EXP: Expansin
GO: Gene ontology
KEGG: Kyoto Encyclopedia for Genes and Genomes
PCA: Principal component analysis
PE: Pectinesterase
PL: Pectinlyase
XTH: Xyloglucan endotransglucosylase/hydrolase
